# Experimental Investigation of Reinforced Concrete Beam with Openings Strengthened Using FRP Sheets under Cyclic Load

**DOI:** 10.3390/ma13143127

**Published:** 2020-07-14

**Authors:** Rania Salih, Fangyuan Zhou, Nadeem Abbas, Aamir Khan Mastoi

**Affiliations:** 1 School of Civil Engineering and Mechanics, Huazhong University of Science and Technology, Wuhan 430074, China; rania_salih@hotmail.com (R.S.); nadeema@hust.edu.cn (N.A.); ak.mastoi21@gmail.com (A.K.M.); 2 Department of Civil Engineering, Quaid-e-Awam University of Engineering Science and Technology, Nawabshah 67450, Pakistan

**Keywords:** cyclic loading, RC beam, FRP strengthening, beam with circular opening, FE modelling

## Abstract

In this study, the cyclic behavior of reinforced concrete (RC) beam with openings strengthened using carbon fiber-reinforced polymers (FRPs) was experimentally investigated. Seven rectangular RC beams were cast and strengthened through external bonding of carbon fiber-reinforced polymer (CFRP) sheets around the beam web opening with different orientations to evaluate the maximum resistance, secant stiffness, strength degradation, ductility, energy dissipation capacity and behavior of the specimens’ failure mode under cyclic load. One solid beam without an opening (i.e., control specimen) and six beams constructed with circular web openings typically located in the middle of the beam and adjacent to the supports were used in the experiments. Among the six specimens with opening configuration, two beams were unstrengthened, and the remaining four specimens were strengthened with two layers of FRP sheets with vertical and inclined scheme orientation. Numerical studies were performed on ABAQUS software, and finite element modelling analysis results were verified through experiments. Results demonstrated that the use of FRP sheets has a significant effect on the cyclic behavior of RC beams, thereby improving the maximum strength and ultimate displacement to approximately 66.67% and 77.14%, respectively. The validated finite element models serve as a numerical platform to apply beneficial parametric studies, where the effects of opening size and bond length are investigated.

## 1. Introduction

The transverse opening requirement in the web of reinforced concrete (RC) beams to allow the passage of services in modern buildings has received significant attention in structural engineering practices, particularly in floor beams [1]. The opening in part of the beam configuration eliminates the amount of self-weight and provides a compact and economical design. However, the appropriate change in the beam cross-section causes high stress concentration in the vicinity of the opening area, thereby affecting the stiffness and shear capacity of the beam [2]. This condition can be addressed by providing a special type of strengthening at the soffit faces, such as the arrangement of adequate internal longitudinal reinforcements or improvement of the external surface area through suffusion quantity of strengthening [3]. Steel plates or fiber-reinforced polymer (FRP) composites are used to restore the original capacity and optimize the damage ability. Although the behavior of a beam with an opening under monotonic load has been widely investigated in structural practices, few explicit provisions or recommended guidelines for the design (ACI318) [4] and limited outlines for the openings are provided in the ASCE-ACI report [5]. Considerable provisions and guidelines were discussed in the previous investigations of Nasser et al. and Mansur et al. [6], who concluded that the opening position must be avoided near the inflection point and that transverse opening causes a reduction in the shear strength of the element, which should be considered in the system.

FRPs have been widely used as alternative materials to strengthen structural elements because of their several advantages, such as low weight, easy installation, high mechanical stiffness and strength, immunity to corrosion and superior resistance to electromagnetic and fatigue load [7,8]. Significant tests have been conducted in the application of FRP sheets for strengthening the concrete elements, and huge success has been achieved in strengthening and repairing the damage or retrofitting for seismic deficiencies [9,10]. Most experimental investigations in the literature have focused on the behavior of RC beam strengthening (without opening) with FRPs, whereas few studies have concentrated on beams with openings. Numerous studies have investigated the behavior of beams with openings and verified the effectiveness of FRPs in strengthening under monotonic loading [11,12,13,14]. These studies show the effectiveness of FRPs in improving the behavior of structural elements, thereby enhancing the load-carrying capacity, demonstrating superior shear, flexural and torsion resistance capacities and enhancing pre-cracking stiffness. These constructive results indicate the good efficiency of using FRPs in different available forms, such as rods, meshes and sheets. Most studies have focused on the behavior of RC with openings under static loading, and few studies have assessed the behavior under cyclic load. [15,16,17]

Herrera et al. [18] investigated the cyclic behavior of T-shaped RC beams with openings. Large-scale experimental specimens (case study) were used to replicate the existing structural beams in California. Four cantilever beams with lengths ranging from 3.8 to 5.3 were constructed. One specimen was a solid beam without opening, whereas the three remaining beams were built with a rectangular web opening of 0.98 m × 0.37 m (length by height). Different reinforcement schemes and opening influences were evaluated to assess the seismic capacity and strengthening mechanism. A hysteretic response curve provided significant information about strength degradation, ductile capacity and energy dissipation. These results demonstrated that additional reinforcement around the web opening prevents premature shear failure in the beam, and the appropriate opening introduced in the beam frame requires careful detailing in the beam region.

Osman et al. [19] experimentally investigated the strengthening of deep beams with openings through the use of a steel plate and evaluated this technique in repairing pre-cracks occurring under a sustained load. Seven beams with circular openings in the shear zone were tested. A temporary pre-stress force was applied prior to steel plate implementation on reinforced beams. They reported that shear strength is obtained by closing existing cracks around the opening through epoxy injection, and the steel plate has significant effects on strengthening and failure mode. An analytical equation was also proposed to predict the shear strength of pre-damage.

Elsayed and El et al. [20] tested 15 deep beams with square web openings retrofitted using Ferro-cement laminates to study the effects of steel wire meshes, thickness and strength of plastering mortar and the opening location in flexural and shear zones with different strengthening schemes on the behavior of deep beams. Their study revealed that the ultimate failure load, ductility ratio and un-cracked stiffness of strengthened beams (in the shear zone) were improved (19–85%).

Hassan et al. [21] evaluated 57 RC beams with a rectangular opening on finite element program ANSYS V12. The modelled beams had a rectangular opening and were externally strengthened with FRP laminates and steel plates. The opening location varied from the beam center to close to the support. Carbon FRP (CFRP) and glass FRP (GFRP) were used in different schemes to analyze and investigate the failure mode, crack pattern, internal strain and energy absorption. The results confirm the experimental work outcomes for these type of strengthening beams at the opening zone using FRP sheets and steel plates. These parameters significantly improve the overall rigidity and partially recover the beam stiffness using FRP strengthening.

Nie et al. [22] used eight full-scale T-section RC beams to evaluate the effects of an induced opening on flexural capacity and to investigate the strengthening in shear capacity. A large rectangular web opening and CFRP U-jackets with a CFRP spike were considered. The specimens were tested under central point loading, and the results showed that the suitable web opening size in the beam configuration can effectively reduce the flexural capacity. CFRP strengthening is necessary to avoid shear failure, and confining the web chord formed by the opening ensures the ductile response for the beam.

Elsanadedly et al. [23] investigated the strengthening effects of FRP on RC beams with rectangular web openings through experiments and numerical simulations. Seven beams with two rectangular opening sizes in a shear zone were tested under two-point loading. Two layers of CFRP sheets and hybrid GFRP with steel plate anchors were used to strengthen the beams in different schemes. Numerical simulation was conducted to validate the experiment result followed by parametric study. Their study classified rectangular openings under monotonic loading as small, large and very large. The accuracy of finite element modelling (FEM) was confirmed in assessing the stiffness and strength of RC beams with large rectangular openings.

There are no experimental investigations reported in the open literature or studies investigating the behavior of RC beams with web openings that are strengthened using FRP materials under cyclic loading. The present study conducted experiments and numerical simulations of RC beams with openings strengthened for shear/flexural deficiency through external bonding of CFRP sheets in the form of u-warp scheme. Seven full-scale specimens were evaluated under reverse cyclic loading through four-point bending. The studied parameters included opening location and strengthening orientations. Numerical simulations were performed on finite element (FE) software ABAQUS-6.14 [24].

## 2. Experimental Program

### 2.1. Specimen Details and Parameters

Seven rectangular RC beams were used, consisting of one solid beam and a further six with openings in different locations. The specimens were tested under reverse cyclic loading. The effects of web opening location and FRP orientation were studied. Three of the tested beams were treated, un-strengthened and considered as control beams. The first beam is solid without an opening, and the remaining beams have a web opening in flexural and shear zones. A circular opening was formed prior to concrete casting using a steel pipe tube. The strengthened specimens contain four beams with web openings. The CFRP sheet was bonded using an adhesive resin material around the opening in u-warp schemes.

**Figure 1:**
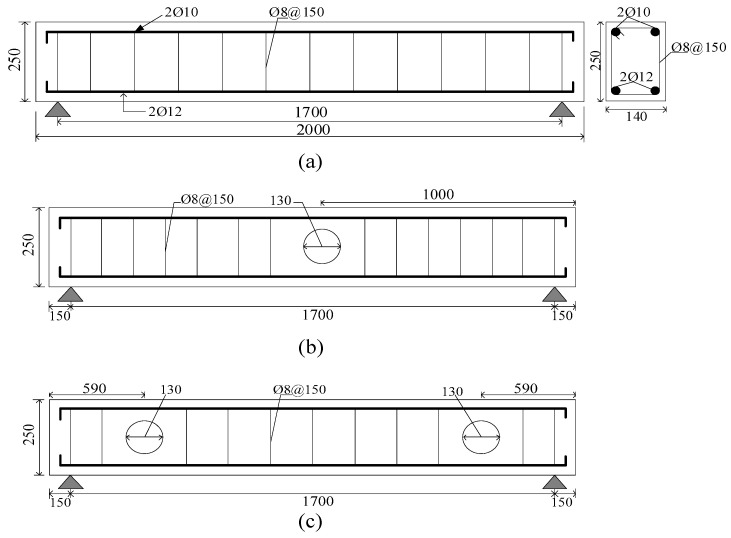
Details of un-strengthened beams (control beams): (**a**) beam dimension and section details, elevation of solid beam B-0, (**b**) elevation of BC-1, (**c**) elevation of BC-2. Note: all dimensions in mm.

All rectangular section beams have the same cross-section with 140 mm width, 250 mm height, a total length of 2000 mm and the same reinforcement arrangement. The longitudinal reinforcement is composed of two 12 mm diameter steel deformed bars in the bottom and two 10 mm at the top of the beam. The shear reinforcement (stirrup), designed to ensure shear failure, consists of 8-mm diameter smooth steel bars spaced at 150 mm to the beam center. All details and dimensions of the tested specimens are shown in Figure 1. The clear span of the beam is 1700 mm, and the effective depth is 220 mm. The clear covers maintained at the bottom and top of the beam are 30 and 20 mm, respectively, whereas 20 mm clear cover is maintained at the two vertical sides. The circular opening has a diameter of 130 mm. The ratio of opening size to the overall beam depth is 0.52, confirming the large opening and ensuring sufficient reduction in the total capacity of the beam. Thus, the efficiency of the strengthening technique can be assessed. The opening has two locations, as directed in Figure 1b,c. The beams were cast in a horizontal position using plywood molds.

**Figure 2:**
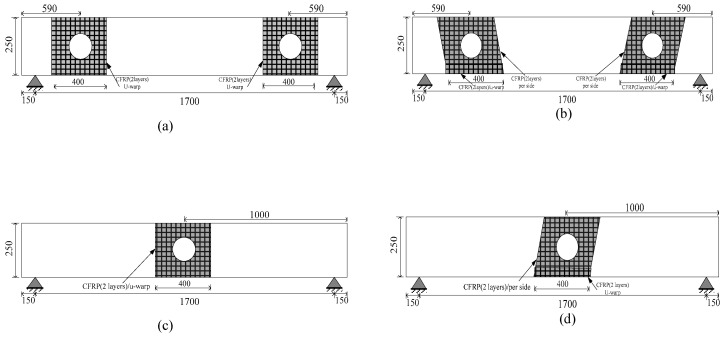
Details of strengthened beams with CFRP orientation: (**a**) vertical u-warp, elevation of BC-3, (**b**) inclined u-warp, elevation of BC-4, (**c**) vertical u-warp, elevation of BC-5, (**d**) inclined u-warp, elevation of BC-6. Note: all dimensions in mm.

Details of all tested beams and various parameters are listed in Table 1. The specimens are denoted from numbers 0 to 6 and letters B and BC, where B refers to the beam, and BC denotes the circular opening beam. Specimen (B-0) refers to the solid beam without opening, and (BC-1, BC-2) denotes the opening beams at different locations (bending and shear zones). The three specimens were considered the control beams and tested to failure without FRP strengthening, as shown in Figure 1. The four remaining beams from BC-3 to BC-6 had a similar opening (shear/bending zone) and were strengthened with externally bonded CFRP sheets. The strengthening process lasted for seven days to ensure sufficient curing of the CFRP epoxy. CFRP was applied in three faces of the beam in the form of u-warp around the opening region and had a consistent 400 mm bond length, 250 mm height and 140 mm width, as shown in Figure 2. The details of the tested beams are summarized in Table 1.

### 2.2. Preparation of Specimens and CFRP Installation

For all the strengthened specimens, the bonded concrete surface was prepared carefully (grinding, smoothening and rounding) before applying the FRP composites. The strengthened region was considered, as shown in Figure 2. Two layers of CFRP sheet were bonded with an epoxy resin. The warped layer had a nominal thickness of 0.338 mm and was applied in two orientations around the web opening vertically and diagonally with 90° (Figure 2a,c) and 60° angles (Figure 2b,d), respectively [25]. The corner of the beam was rounded with a radius of 10 mm to avoid stress concentration. The circular cut in the CFRP sheet was accurately created in accordance with the web opening size. The cutting in vertical direction (90° angle) was smooth during practical application. For inclined orientation, every side was separately applied to ensure continuity and prevent any delamination shear. U-warp with 400 mm length, 60 mm height and 140 mm width was used and bonded to the bottom chord of the concrete below the opening. The inclined fabric sheet was bonded to the two sides of the beam. CFRP strengthening was applied through the wet-layup process, which includes the following steps [26]. Firstly, surface dust was removed with an air blower. Then, a layer of well-mixed primer epoxy was applied (CFSR-A, CFSR-B), according to manufacturer’s instruction for mixed percentage, and stirred well to reach homogenous consistency, followed by concrete surface painting using a paintbrush. Carbon sheets saturated with well-mixed epoxy resin CFSR-A and CFSR-B were laid on the concrete surface. Finally, FRP was slowly rolled to release any air bubbles entrapped between the CFRP layers and concrete to achieve regular distribution of epoxy resin. The specimens were cured for seven days under laboratory conditions prior to testing [27].

### 2.3. Material Properties

The properties of the materials, particularly the concrete and steel, used in this study were experimentally obtained. Twenty-one specimens with dimensions of 150 mm × 150 mm × 150 mm were cast and subjected to the same curing conditions and composition properties (of beam element) to ensure a similar compressive strength to that of the natural one to determine the compressive strength of concrete. The compressive strength results were obtained after testing the cubesusing a compressive testing machine. The average test results of concrete strength after 28 days were approximately 30 MPa. To determine the tensile strength of steel bars, the standard specifications based on B.S. (British stander) were considered and reviewed to meet the G.B. (Chinese code of practices) code. The yield and tensile strengths were obtained by testing three bars with diameters of 12, 10 and 8 mm, respectively, using a tensile testing machine, as shown in Table 2. For the CFRP used in this study, the material properties were provided by the manufacturers, as shown in Table 3. CFRP sheets have a tensile strength of 3400 MPa and elastic modulus of 230 MPa. The detailed properties of the CFRP sheet and epoxy resin are shown in Table 3.

### 2.4. Setup of Test Models

**Figure 3:**
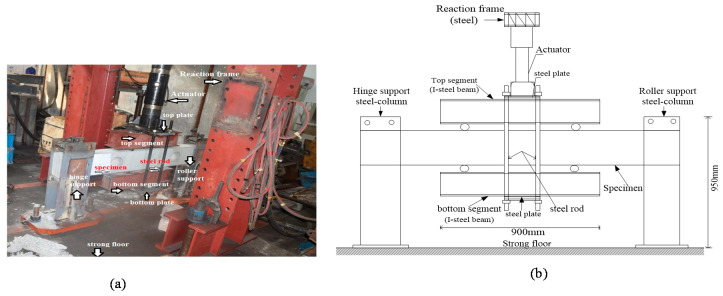
Experimental setup of tested beams: (**a**) photograph of test setup for BC-2. Note: the other beams were similar to that shown. (**b**) Schematic test setup for all beams.

**Figure 4:**
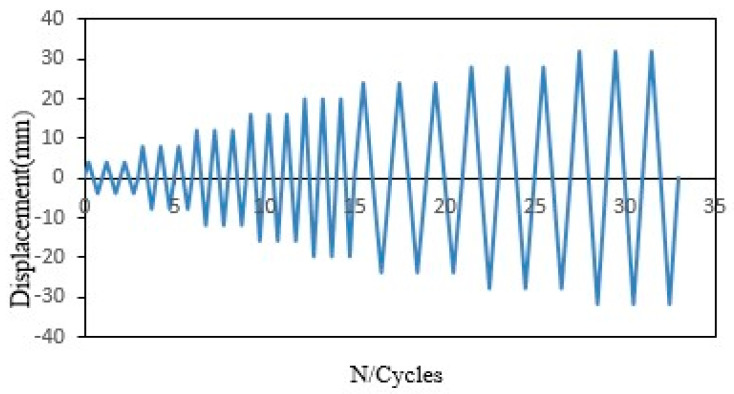
Imposed load history.

The schematic and photograph (side view) of the test setup and the arrangement of measurement pieces of equipment and other components are shown in Figure 3. The specimens were tested on a rigid platform on the laboratory floor, consisting of two steel column supports with a height of 80 cm < h < 95 cm. The specimens had a boundary condition as simply supported with mounting. All beams were tested under four-point bending in the form of cyclic loading using a hydraulic actuator [28]. The reaction portal frame with 3.5 m height, 1.8 m width and massive weight steel was fastened on the laboratory floor and secured in front of two supports of the beam to prevent any rotation and sliding. Cyclic loading was applied using a hydraulic jack that conveyed the load from a 300 kN capacity to the top middle of segments (I-section steel beam). As shown in Figure 3, the top and bottom of the tested beams were clamped using two segments with rollers and four studs attached at 0.25 length to the beam surface to control and distribute the forces; the distance between the concentrated forces is 500 mm. This setup allowed reverse cyclic loading on the beam while maintaining its freedom to rotate at one side, which provides a good way to simulate seismic action [29]. The beams were subjected to impose load history, which contains reversed controlled displacement and gradual compressive axial load. Each cycle was repeated twice, where the first cycle was initiated with +1 mm, the second cycle increased to +2 mm, and the remaining cycles increased by +2.5 mm in each cycle until reaching 33 mm, as shown in Figure 4. Mechanical load was utilized using a capacity hydraulic actuator. Various signals from instruments (measurement of load change and displacement) were captured and observed using an automated digital system. The controlled displacements were measured using linear variable differential transducers (LVDT).

## 3. Experimental Results and Discussion

### 3.1. Failure Mode and Crack Patterns

**Figure 5:**
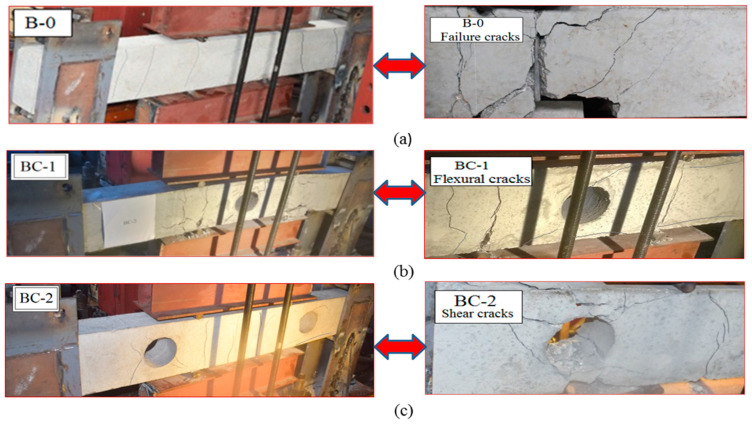
Observed failure mode for unstrengthen beams: (**a**) solid beam B-0, (**b**) BC-1, (**c**) BC-2.

**Figure 6:**
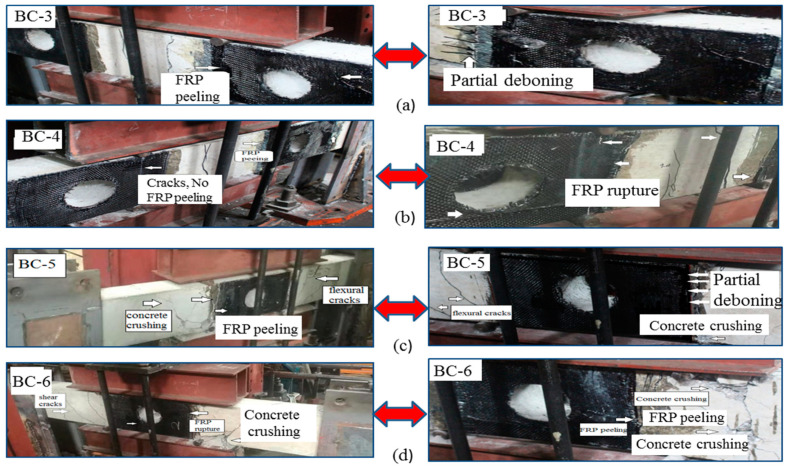
Typically observed failure mode for strengthened beams: (**a**) BC-3, (**b**) BC-4, (**c**) BC-5, (**d**) BC-6.

Photographic summaries that display the performance of the tested beams under various load levels are presented in Figure 5 and Figure 6. The key results are summarized in Table 4. No crack formations are observed on all specimens at the initial displacement level. With the increase in displacement amplitude and load sequence, fine cracks are formed and continuously enlarge. The cracks propagate on the beam surface at the shear span and bending regions and continue to gradually increase until reaching failure. The appearance of cracks was usually observed at the vertical position close to the left part of the constant moment zone at a displacement ranging from ±7.5 mm to ±15 mm actuator load. Corresponding to the same level, the first cracking loads developed at 35% of the yield strength of the beams.

Figure 5 shows the crack and failure patterns of the beams during the application of loads until reaching total damage. Specimen (B-0) is a solid beam without opening. Typical classical cracks appeared at 42.55 kN load level caused by the yield of tension repair after the beam developed vertical cracks. The main cracks localized between the load points and supports. Widening in the existing cracks and degradation of the concrete are observed near the load point with the increase in displacement level to ±15 mm. Then, the beam failed because of concrete crushing in the form of flexural failure at the maximum moment region. For specimen (BC-1) with an opening in the flexural zone, the early cracks formed close to the applied load region and expanded to the shear span. The flexural cracks, including vertical and inclined cracks, appeared relatively unaffected because of the presence of the opening. However, longitudinal reinforcements did not yield, and no considerable damage occurred. The first crack formed under the load center point from the bottom to the top direction with the increase in displacement level rising to ±12 mm at 38.75 kN load level. The diagonal cracks (approximately 45° to the longitudinal direction) passed through the opening center and stopped without a progressive increase in size. The flexural and shear cracks enlarged during loading, thereby causing concrete crushing. Subsequently, specimen failure occurred because of flexural cracks. For the beam with an opening in the shear zone (BC-2), the increase in load and displacement levels at ±10 mm caused the propagated diagonal cracks to pass through the opening center near the roller support. The first crack appeared on the beam under the direct point plate at the 34.25 kN load level. Many cracks appeared along the beam surface with continuous loading. As shown in Figure 5c, wide cracks appeared at the bottom and top chords (both chords) around the beam opening, thereby causing concrete spalling and crushing, and additional vertical cracks formed at the bending region. The specimen failed because of shear cracks in the opening region.

Figure 6 shows the crack patterns and failure mode of the strengthened beams. The external bonding interface of CFRP was considered to enhance the weakest area around the opening. For specimens (BC-3) and (BC-4,) the failure is identified by the initiation and propagation of shear and flexural cracks. The main cracks are concentrated and close to the middle of the beam. At the same time, the first cracks are formed in this region at ±15 mm displacement. With the progressive increase in loading, vertical cracks and diagonal cracks widely propagated in the bending zone, resulting in concrete crushing caused by continuous opening and closing of flexural cracks. As shown in Figure 6a,b, partial de-bonding of CFRP sheet wrapping from the identical location of massive shear cracks accompanied with small audible sounds from the fracture resin was observed prior to the final failure of specimen (BC-3). The first cracks appeared at 47.62 kN and ±15 mm displacement. The widening of the two main cracks in the bending zone was observed because of the continued loading sequence. Hence, the beam failed because of these flexural cracks. Specimen BC-4 developed a crack pattern and failure mode similar to specimen BC-3. The distribution of most cracks at all parts of the beam around the opening in the shear zone and flexural region was observed. The diagonal shear cracks around the opening were evidently captured by the CFRP sheet. No de-bonding failure occurred between the beam surface and the CFRP sheet. As previously mentioned, two layers of CFRP with inclined orientation were used in the concrete surface of this specimen. The application system was improved to capture many diagonal cracks observed in BC-4.

For the two specimens (BC-5 and BC-6), the opening was presented in the flexural zone in the middle of the beam and strengthened using a vertical and inclined u-warp of CFRP. The diagonal cracks firstly appeared on the left side of the web opening (the side nearest to loading point) and extended to the support at ±16 mm displacement and 60.20 and 61.75 kN. The hair cracks propagated in the right side of the opening, and large diagonal cracks appeared at the two sides of the shear span (u-strengthened areas) with the progress in displacement and load sequence. Subsequently, the final failure occurred because of local mixed shear and flexural cracks along with concrete crushing. The shear/flexural cracks around the top and bottom chords of the web opening were prevented because of the existence of the FRP sheet. In specimen BC-5, the partial dependence for CFRP appeared near the ultimate displacement level. The effect of bearing stress on the contact surface between the steel plate and concrete increased the separation, thereby contributing to the final failure. For specimen BC-6, the CFRP ruptured because of the downward and upward loading. This demonstrative strengthening increased the peeling probability at the main fibers and contributed considerable flexural capacity to the specimen. A predominant reduction in crack propagation was observed in the strengthened specimens at their opening locations, particularly in the shear and flexural zones. Furthermore, the usefulness of the epoxy resin layer in the FRP composite was verified.

### 3.2. Hysteretic Behavior

**Figure 7:**
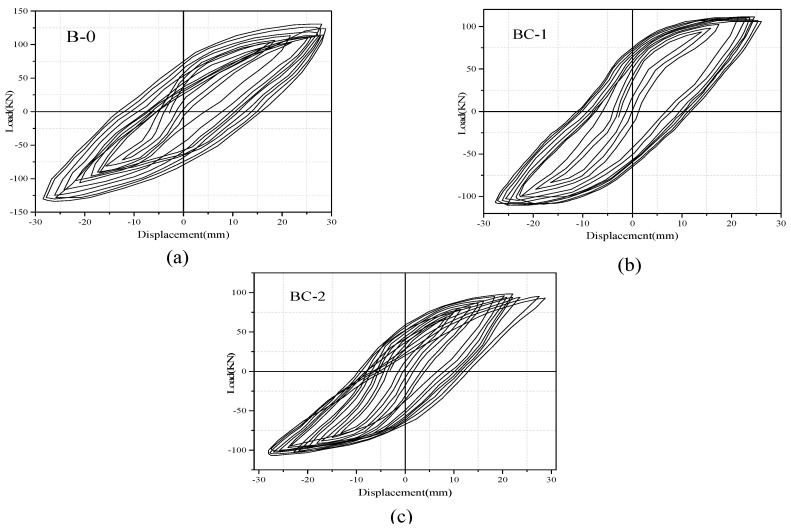
Hysteretic curve of tested specimens: (**a**) solid beam, B-0, (**b**) beam with opening in shear zone, BC-1, (**c**) beam with opening in flexural zone, BC-2.

**Figure 8:**
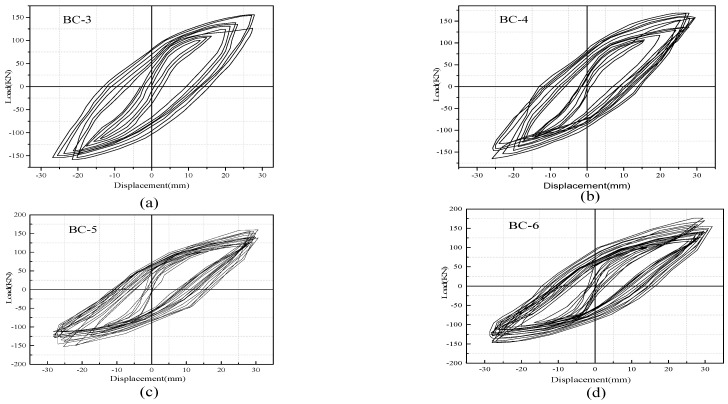
Hysteretic curve of strengthened specimens: (**a**) BC-3, (**b**) BC-4, (**c**) BC-5, (**d**) BC-6.

The hysteretic response of each specimen was presented in the form of load–displacement (P–Δ) relationships, as depicted in Figure 7 and Figure 8. The measured results show the performance and failure phenomena of the beams. The positive and negative values of load and deformation correspond to the downward and upward load directions. All the specimens displayed full hysteretic loops to ensure adequate inelastic behavior. With the increase in displacement rate (in the imposed protocol), the load capacity of the specimens decreased during two to three successive completed cycles with similar a repetition level, and the hysteresis loops changed their direction from a straight elastic line to an inelastic curvature. The cracks opened and the load-carrying capacity significantly deteriorated when the beams were pushed. However, the shear cracks opened, and the stiffness (slope of the curve) immensely decreased when the beams were pulled down. This finding is because of the decay of the concrete and degradation of load-carrying capacity. Three major levels were noted in the response of specimens, documented and recorded during the test, and were used to evaluate the strength and stiffness. The first level represents the un-crack level, the second level denotes the ultimate equivalent to the yield strength and the third level indicates the peak level. Max strengths and corresponding ultimate displacement prior to failure P_max_ and −Δ_max_ are defined on the basis of the point of maximum experimental strength. Yield displacement Y_max_ is considered the maximum displacement in the plastic range and can be extracted from the corresponding peak displacement when the ultimate load is decreased to 80% of the maximum strength, as listed in Table 4. Secant stiffness (K_i_) is studied in a positive loading range and is calculated as the ratio between the yield load and max displacement in the plastic range. The ductility capacity of the specimens (μ) is an important index used for seismic responses of RC structures, which is measured in terms of displacement capacity and evaluated by dividing the peak-to-yield displacement.

**Figure 9:**
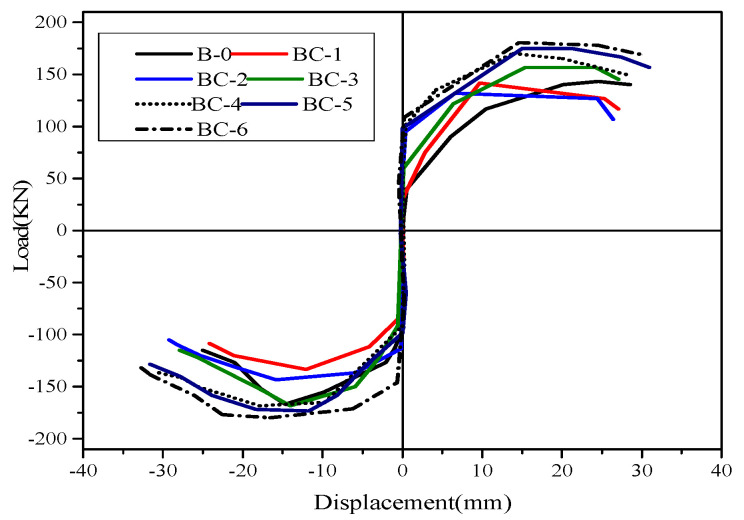
Strength envelopes for unstrengthened and strengthened beams.

For the comparison of hysteretic behavior, the measured loads versus displacement envelop curves of unstrengthened and FRP strengthened specimens are plotted and compared, as shown in Figure 9. The analysis of hysteretic behavior in all specimens shows that a small displacement level has no significant effect on hysteresis performance, whereas large displacement has considerable response. In accordance with the load versus displacement envelope response, the stiffness variations of each beam can be obtained as the ratio between the applied load and displacement and determine the load that causes failure. Specimens BC-1 and BC-2 have lower loads than those of the loads supported by solid specimens (B-0), which has the maximum load of 118.92 kN and displacement (28 mm) because of the presence of the opening. Specimens BC-1 and BC-2 reach maximum loads of 98 and 94 kN, respectively (displacements of 26 and 24 mm). Thus, the percentage of load-carrying capacity decreases by 16.89% and 20.61%. The strengthened specimens reach the greatest load with a mean value of 157.37 kN and a displacement of 30 mm for specimen BC-3, and 172.81 kN and a displacement of 32 mm for BC-4. This value is 66.69% higher than that of the BC-2 specimen and 74.84% higher than that of the BC-1specimen. The use of a carbon fiber sheet is highly effective in increasing the maximum strength and ultimate displacement for beams with openings in the shear/bending zone and improves the beam ductility.

### 3.3. Secant Stiffness and Strength Degradation

**Figure 10:**
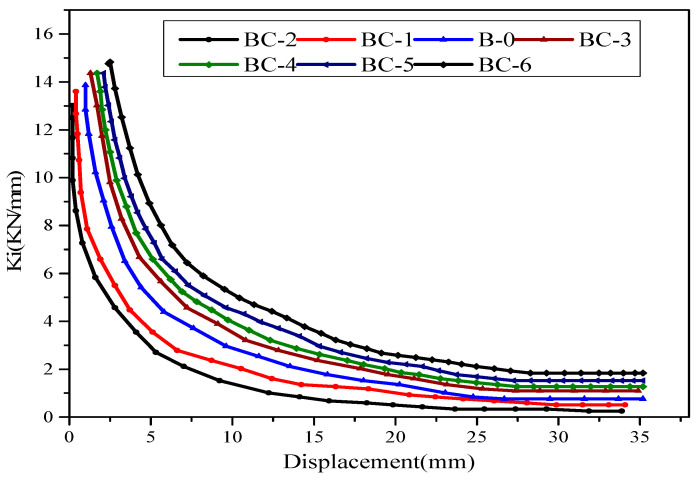
Stiffness degradation for unstrengthened and strengthened beams.

During cyclic loading, the specimens exhibited stiffness and strength losses. This phenomenon is considered a good parameter to measure the structural decay with increasing displacement rate and cyclic numbers. In this study, the deformation restoring capacity affects the beam stiffness thereby resulting in crack propagation, reinforcement and peeling or rupture of the FRP. Stiffness loss increases at a variable rate with the increase in maximum displacement, as indicated by the decrease in the slopes of the load-displacement hysteresis loops (caused by open cracks) at pinching of the elastic loop. The stiffness of the beams at a certain displacement level is considered the average stiffness in two (positive/negative) loading directions. It is computed as the ratio between the peak load of the loop and the associated displacement. Secant stiffness versus displacement is plotted in Figure 10. The response of the specimen shows that stiffness loss degrades more in the opening region of specimen BC2 than that in BC1, which is attributed to the concrete deterioration close to the opening in the shear zone. Towards the last level of the loading cycle, the stiffness for two specimens is relatively low. For specimens BC3 to BC6, the effect of the strengthening technique improves the stiffness degradation compared with the reference specimen. The initial stiffness is approximately the same for all beams. In all strengthened specimens, a good increase in stiffness is found during the first few cycles. Hence, the strengthening technique used in this study increases the strength values of the specimens. Subsequently, a progressive decrease in stiffness and strength (load-carrying capacity) is observed with the increase in displacement rate and subsequent cycles. As previously explained, stiffness is calculated as the ratio of the maximum load of the loop to the associated displacement for positive values. The secant stiffness degradation rates for all the beams ranged from 15.49% to 46.34%. The increase in stiffness at every displacement rate for specimens BC-3, BC-4, BC-5 and BC-6 is significant, suggesting that the two layers of CFRP around the opening contribute to stiffness. On the contrary, intense stiffness degradation is found until the peak displacement level is reached for the unstrengthened beams. The stiffness degradation of the specimens with the opening in the shear zone is higher than that in bending. The change in FRP from vertical to inclined orientation improves the stiffness degradation for specimens BC-4 and BC-6 and it is clearly effective in BC-4. This enhancement is attributed to the 60° inclined CFRP sheet, which is vertical to the angle of the diagonal shear cracks. Therefore, this condition can promote the concrete property that effectively captures the shear cracks and partially optimizes the ductility of the specimens.

### 3.4. Absorption and Energy Dissipation

**Figure 11:**
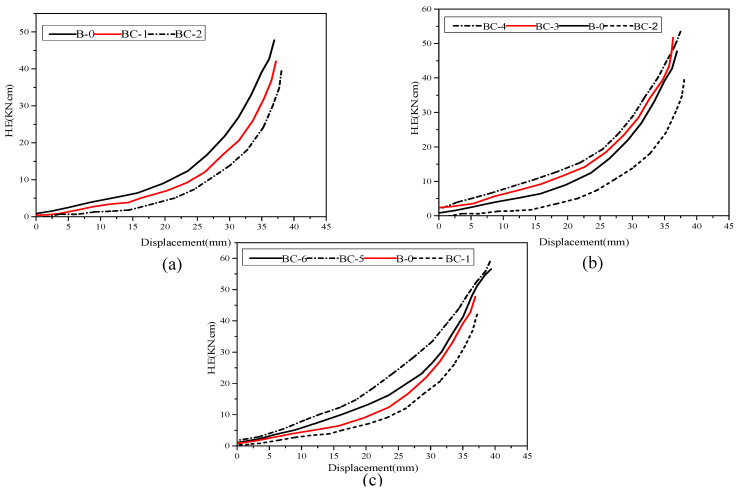
Energy Dissipation for tested specimens: (**a**) for unstrengthened beams, (**b**) for strengthened opening beams in the shear zone, (**c**) strengthened opening beams in the flexural zone.

The capacity dissipating energy is an important factor used to evaluate the performance of structural elements subjected to cyclic action. The structure resistance strongly depends on the absorption and dissipation of the seismic energy force. The energy dissipated by the beams during the individual cycle is measured as the area enclosed within the load-displacement hysteresis energy loop [30]. Then, the overall energy dissipated is computed as the summation of the areas of the loops throughout the obtained test results. It is estimated through numerical integration for the listed load times to the displacement. The normalized hysteric energy, NHE, at a given cycle (i) is expressed as follows:

(1) $$NHE_{i}=\frac{\sum_{j=1}^{j=i}HE_{j}}{2(0.5Pu\times \Delta y)}=\frac{HE_{1}+HE_{2}+\cdots +HE_{i}}{\left(Pu\times \Delta y\right)}$$

where *HE_i_* is the hysteretic energy dissipated during the *i*th cycle, ∆*y* is the yield displacement of the specimen, and *Pu* is the ultimate load. The *NHE_i_* dissipation with respect to the applied displacement level is presented in Figure 11.

As shown in Figure 11, the energy dissipating curves of the beams are nonlinear. The amount of energy absorption decreases with the increase in displacement rate in the plastic zone. No considerable difference is found between the hysteresis energy at low displacement. However, the slope in the curve moves upward with the increase in displacement level, indicating a significant improvement in the absorption energy for the specimen. As shown in Figure 11a, the cumulative energy dissipation capacity is relatively decreased in specimens BC-1, BC-2 and B-0 and exhibits a low rate because of the effect of the opening, which is associated with the corresponding reduction in the hysteresis loop. The energy dissipation capacity of these beams decreases by 23.21% and 28.55%, whereas the energy absorption of specimens strengthened with CFRP increases by approximately 64.87% and 77.14%. This finding demonstrates that high bearing capacity and no degradation or strength loss during loading cycles decrease the pinching control of hysteresis loops. These observations confirm the fundamental influence obtained from the material properties of CFPR, in particular, the linear elastic behavior up to tension failure. The hysteretic energy dissipation of strengthened specimens BC-4 and BC-3 has a small variation in energy apportion progress because of the effect of CFRP orientation. The same performance is observed for beams BC-6 and BC-5.

## 4. FEM

FEM was conducted for the development and validation of nonlinear models to simulate the tested specimens and to verify their applicability. The models were generated on commercial software ABAQUS 6.14. Numerical analysis was implemented for seven variable beams subjected to four-point bending, and quasi-static loading was applied. The geometric details, material properties and actual elastic-plastic and stress-strain relationships were obtained from the experiment. The failure modes and hysteretic curves were also analyzed. The following section provides details of the FE procedure.

### 4.1. Model Geometry and Mesh

**Figure 12:**
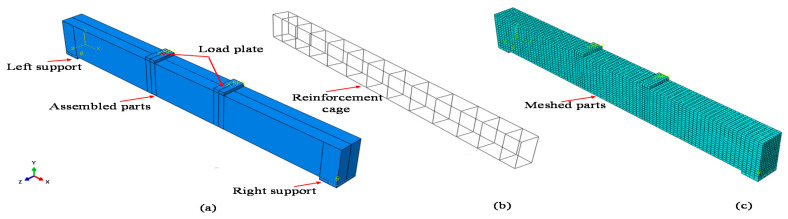

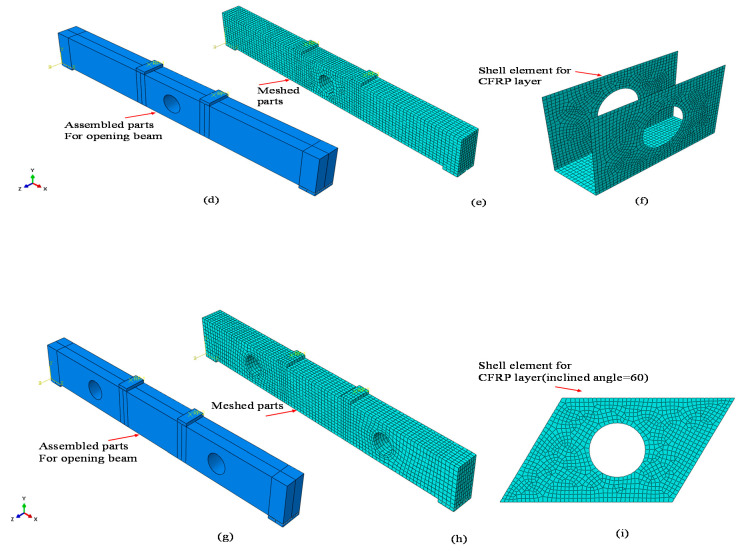
Finite element (FE) models for 3D specimens: (**a**) assembled beam element B-0, (**b**) steel rebars for beam B-0; (**c**) mesh element for B-0, (**d**) opening beam in the flexural zone BC-1, (**e**) mesh elements for BC-1, (**f**) two layers of CFRP for beams BC-3 and BC-5, (**g**) opening beam in the shear zone BC-2, (**h**) mesh element for BC-2, (**i**) two layers of inclined CFRP for beams BC-4 and BC-6.

Three-dimensional model schemes of FE analysis were utilized to simulate the RC beam with an opening and strengthening through external bonding of an FRP sheet. All the components, the concrete beam, steel bars, stirrups, CFRP sheet, and steel plate, were simulated and modelled, carefully to obtain accurate results with a sensible computational time. The element types and mesh size for every part was selected perfectly. Concrete and filling materials were used in a linear eight-node 3D solid element with reduced integration (C3D8R) and had three degrees of freedom per node, thereby providing ideal handling for most applications whether in linear or complex nonlinear analysis, including contact, plasticity and large deformations. Longitudinal reinforcement is defined as the truss element with three translational degrees of freedom (T3D2) at each node. The composite material CFRP was formed in a 3D solid deformable element at four-node thin shell element reduced integration (S4R) [31], with three degrees of freedom per each node, and tied to the surface of three sides of the beam. The mesh configurations of the specimens are considered to conduct the convergence study, as shown in Figure 12. The concrete beam was meshed using an eight-node solid hexahedron element. The entire mesh size was refined for the specified area around the circular opening. A two-node truss element type with linear geometric order was used to mesh the steel bar and stirrup, whereas a four-node quad-dominated shell element was used for the CFRP sheet, as seen in Figure 12, and the steel plate was meshed using an eight-node solid hexahedron element shape. The approximate global size for the mesh elements varied from 5 mm to 25 mm. A perfect bond was assumed through the use of an embedded region in the FE constraint between the reinforcement cage and surrounding concrete. The external bond between the concrete and FRP was assumed perfect through the use of tie interface action.

### 4.2. Material Model

The properties of the materials were extracted from the experiment, as shown in Table 2 and Table 3, and defined in FEM. The inelastic behavior of the concrete was represented using the concept of concrete damage plasticity (CDP) to define the plastic properties in compression and can be used as the softening behavior either in compression or in tension, thereby simulating the material properties of concrete realistically [29]. Several models have been developed in the literature to calculate and adjust the stress-strain curve during the compression of concrete. Hognestad’s model was chosen in this study [31]. The compressive and tension stresses at any point can be defined using Equations (2) and (3). The bilinear stress-strain relationship response of steel reinforcement was modelled using an isotropic elastic-plastic model. In this procedure, the steel remains elastic up to the yield strength, where plastic deformation continuously occurs. The material properties of steel reinforcement applied during the experimental work are summarized in Table 2. The absolute elastic case was used to simulate the behavior of CFRP composite materials. In this case, the behavior of FRP sheets was assumed to be linear until reaching the plastic strain, where cracks extend, and the material loses their load-carrying capacity at the same time. The characteristics used are listed in Table 3.

(2) $$\sigma_{c}=\left\{ \begin{array}{l} f_{c}\left[2\frac{\varepsilon }{\varepsilon_{0}}-{\left\{\frac{\varepsilon }{\varepsilon_{0}}\right\}}^{2}\right]\\ f_{c}\left[1-0.15\frac{\varepsilon -\varepsilon_{0}}{\varepsilon_{\mu }-\varepsilon_{0}}\right] \end{array}\right.$$

(3) $$\sigma_{t}=\{f_{t}\left[1.2\frac{\varepsilon }{\varepsilon_{0}}0.2{\left\{\frac{\varepsilon }{\varepsilon_{t}}\right\}}^{6}\right],f_{t}\left[\frac{\frac{\varepsilon }{\varepsilon_{t}}}{a_{t}{\left(\frac{\varepsilon }{\varepsilon_{t}}-1\right)}^{1.7}+\frac{\varepsilon }{\varepsilon_{t}}}\right]$$

where *σ_c_*, *σ_t_*, is the compression and tension stress, *f_c_*, *f_t_* is compressive and tensile strength, *ε* is strain, and *ε*_0_, *ε_u_* is yield and ultimate limit strain.

### 4.3. Loading and Boundary Condition

The beam models were subjected to slow cyclic loading on their top face, which was simulated as the actual loading setup in the experiment. The beam was tested under four-point bending (as shown in Figure 3). Steel bearing plates with sizes of 140 mm × 70 mm × 20 mm and 140 mm × 100 mm × 20 mm were placed under the point loads and at two rigid supports, respectively. The tie condition was numerically implemented to simulate the constraint between the plates and the specimen. A reference point in the middle of each plate was used to apply the cyclic load and was connected to the beam through the coupling constraints provided in ABAQUS. The numerical models of each specimen were created with the same condition as in the test. Then, three boundary conditions were created, two of them for the initial step and one for step one. For the modelled rigid supports, the left side acts as pin support to allow rotation in only one direction and provide restraint to lateral displacement, and the right side acts as roller support to allow the beam to rotate freely (U_x_ and U_y_ are restrained). Load was applied at two loading points as the vertical displacement (at the third boundary condition), and the amplitude data were obtained in the same step. The rate of maximum increment time was increased to minimize the solution time to represent the quasi-static load in implicit analysis.

### 4.4. Failure Model

The failure model available in ABAQUS is based on the model developed by Lubliner et al. [32] and has been modified by many researchers. The predictions by Lee and Fenves [33] were selected in this study. The model can directly predict the failure of concrete by creating and defining the CDP in constitutive relationship material behavior. As previously mentioned, part of the test result shows that the failure observed through small cracks starts appearing at the beam section and increases and coalesces until failure occurs. The plasticity behavior can be described by several phenomena, such as strain softening, gradual deterioration and volumetric expansion, thereby causing a reduction in strength and stiffness of concrete [34]. The stiffness degradation assumes that the failure mechanism is related to the accumulation of two factors, namely, tensile cracking and compression crushing of the concrete material. Additional details of the CDP model, relevant background theories and actual application strategies can be found in the ABAQUS Analysis User’s Manual [24]. The plastic stress-strain for concrete damage is obtained through the numerical expressions in Equations (4) and (5) to evaluate plastic damage when degradation occurs only in the softening range and the stiffness is proportional to the cohesion of the material.

(4) $$\frac{E}{E_{0}}=\frac{C}{C_{\max}}=1-d$$

(5) $${\bar{\varepsilon }}^{p}=\varepsilon^{p}-\frac{d}{1-d}\frac{\sigma }{E_{0}},\;and\;{\bar{\varepsilon }}^{p}=\varepsilon -\frac{f}{E_{0}}$$

where *C* is the cohesion in the yield criteria, which is proportional to stress, *C*_max_ is equivalent to the strength of the concrete, *d* is a damage factor, and *f* is either the tensile or compressive strength of concrete and can be related to the damage factor *d*, and *ε^p^* is plastic strain damage.

The damage compression for model elements and tension parameters is estimated using Equations (6) and (7), respectively [31].

(6) $$d_{c}=1-\frac{\sigma_{C}E_{C}{}^{-1}}{\varepsilon_{c}^{pl}\left(\frac{1}{bc}-1\right)+\sigma_{c}E_{C}{}^{-1}}$$

(7) $$d_{t}=1-\frac{\sigma_{t}E_{C}{}^{-1}}{\varepsilon_{t}^{pl}\left(\frac{1}{bt}-1\right)+\sigma_{t}E_{C}{}^{-1}}$$

where *σ_c_* and *σ_t_* are the compressive and tensile stresses, respectively, *E_c_* is the modulus elasticity of concrete, $\varepsilon_{t}^{pl}$ is the plastic strain corresponding to *σ_c_* and *σ_t_*, and *b_c_* and *b_t_* are constants in the range of 0 < *b_c_*, *b_t_* < 1. The values of *b_c_* and *b_t_* are proposed by [35] and equal to 0.7 and 0.1, respectively.

## 5. Verification of Numerical Modelling

**Figure 13:**
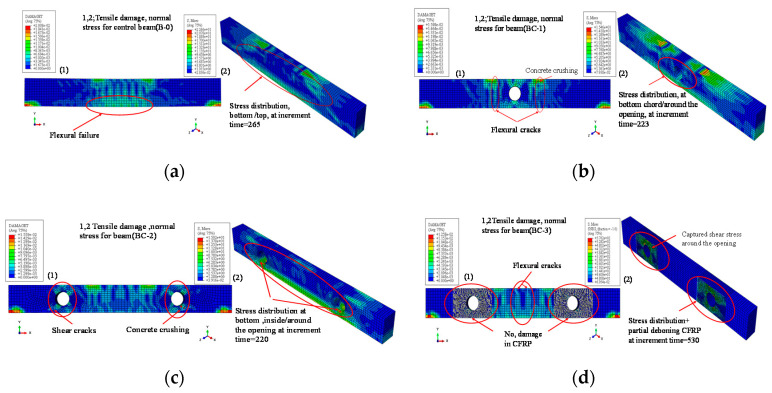

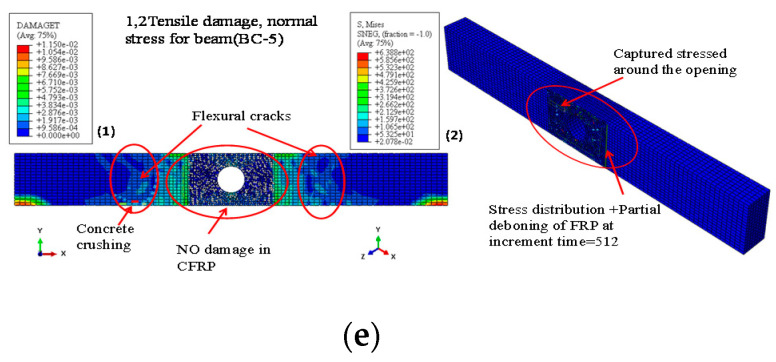
FE result for mode failure of specimens, comparison with the experimental results: (**a**) B-0, (**b**) BC-1, (**c**) BC-2, (**d**) BC-3, (**e**) BC-5.

**Figure 14:**
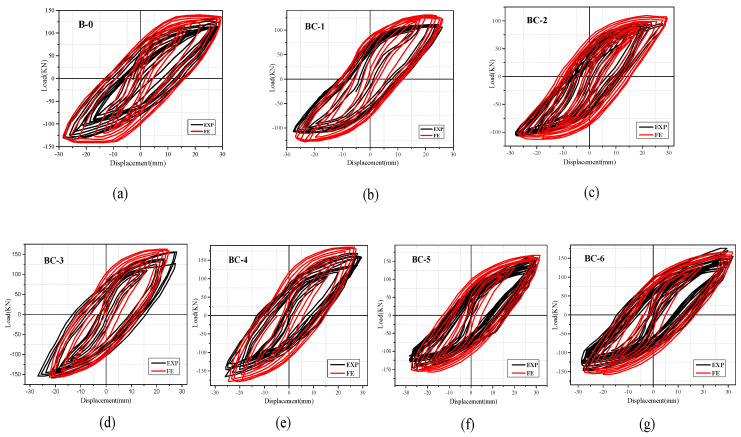
Comparison of hysteretic curve for experiment and FE: (**a**) B-0, equivalent M1, (**b**) BC-1 equivalent M2, (**c**) BC-2, equivalent M3, (**d**) BC-3, equivalent M4, (**e**) BC-4, equivalent M5, (**f**) BC-5, equivalent M6, (**g**) BC-6, equivalent M7.

The results of seven experimented specimens was used to validate the FEM. The modelling result and comparison are summarized and shown in Table 5. The result of FE analysis is explained and discussed in the following subsection in terms of failure mode, as shown in Figure 13, and hysteretic response in Figure 14.

The crack pattern behavior obtained in FEM was represented through the contours of tensile damage and normal stress distribution, as displayed in Figure 13 for some modelling results. These figures clearly show that the failure modes are relatively similar to those recorded in the experiment. Numerical analysis shows that control beam (B-0) fails in a semi ductile manner because of concrete crushing after the propagation of flexural cracks at the bending zone and stress distribution at the bottom and top of the beam, as illustrated in Figure 13a. For the beam with an opening in the bending zone (BC-1), the flexural cracks accrue around the opening in addition to the distribution of principal stress at the bottom and top chord of the beam, as shown in Figure 13b. The diagonal cracks and vertical cracks cause the failure of the opening beam in the flexural mode, which agrees with the test result. For the beam with two openings in the shear zone (BC-2), the vertical and inclined cracks appear under the point load and are distributed to all parts of the beam. The shear cracks and stress diagonally pass through the opening, and concrete crushing is accrued, as shown in Figure 13c. The final failure mode is because of the shear cracks in the opening region, as previously mentioned. For the beam with a strengthened opening in the shear zone (BC-3), high values of shear stress distribution are obtained around the opening because of the use of the CFRP sheet, as shown in Figure 13d. The specimen fails in flexural mode in addition to partial de-bonding for the FRP sheet on the side. For the beam with a strengthened opening in the bending zone (BC-4), the stress around the opening is well covered. The flexural cracks develop to concrete crushing, thereby causing beam failure in the flexural mode.

## 6. Parametric Study

**Figure 15:**
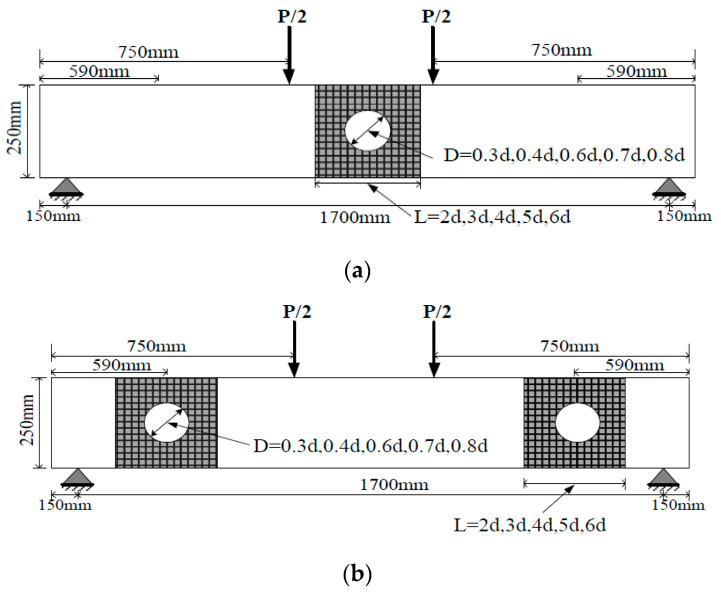
Details of RC beams with openings used in parametric study: (**a**) for group A; (**b**) for group B.

Twenty-nine models of RC concrete beams were used in the parametric study of FE analysis to investigate the effects of FRP sheet bond length and opening size. The studied beam consists of groups A and B; here, A represents the beam with an opening in the bending zone, and B denotes the beam with an opening in the shear zone, as shown in Figure 15. The details of the models used and the results are summarized and shown in Table 6.

### 6.1. Effect of FRP Sheet Length

External bonding by the wrapping of FRP sheets provides a successful method for the retrofitting or strengthening of RC elements subject to either static or seismic loads. The portion of the fiber sheet behaves as a confined-pinned because three sides of the FRP were used to bond the top and bottom chords of the opening [36]. U-warp was used in the beam elements to achieve the required confinement for resisting the tensile peeling stresses and longitudinal crack propagation. The bond length and number of layers had a significant effect on total strength, which was gained from using the FRP sheet. To simplify the calculation, the effective bond length can be estimated from the ultimate contribution of the FRP sheet to the shear strength of the RC beam on the basis of the orientation and crack pattern in (ACI-440.2R-08, 2008) [27]. The effective area can be obtained from the contribution of shear strength for FRP. Khalifa et al. [37] determined the bond reduction coefficient and effective length, which can be expressed as Equations (8) and (9):

(8) $$k_{v}=\frac{k_{1}k_{2}L_{e}}{11,900\varepsilon_{fu}}\leq 0.75$$

(9) $$L_{e}=\frac{23,300}{{\left(nt_{f}E_{f}\right)}^{0.58}}$$

where *k_v_* is the bond reduction coefficient that is a function of concrete strength, type of wrapping scheme used and the stiffness of the laminate, and *k*_1_ and *k*_2_ are the modification factors applied to *k_v_* to account for the concrete strength and bond area. *L_e_* is the effective length.

The above equations provide the estimation value of effective bond length for the FRP sheet and evaluate the strength obtained through the defined layer. The validated FEM was extended to study the effects of the increase in the proposed sheet length on the behavior of strengthened RC beams for groups A and B. In this regard, the value of sheet bond length is directly obtained as L = (n) × (d), where n = 0,1,2,3,4,5,6, d is referred to as the diameter of the opening, and the length used is shown in Figure 15. For groups A and B, 10 models of beams were used and named as B1-1 to B1-5 for group (A) and B2-1 to B2-5 for group (B), as shown in Table 6. The FE results of these models are summarized in the same table.

### 6.2. Effect of Opening Diameter

The effects of different opening diameters on the behavior of unstrengthened and strengthened beams are discussed in this section. Mansur [3] indicated that openings with circular, square and semi square shapes in the cross-sections of flexural members are classified on the basis of the diameter (width)-to-overall depth ratio. Approximately less than 40% is considered a small opening, which follows the similar behavior to that of a solid beam under monotonic loading. In this research, the diameter of the opening is a varied percentage (30%, 40%, 60%, 70% and 80%) of the overall depth. The corresponding distance from the support is unchanged and not used in the parametric study. In general, the suggested opening distance from the support must be greater than 50% of the overall beam depth, for safety [3]. The model details and geometric shapes are shown in Figure 15 and Table 6. Considering that the two layers of U-warp (0/90) are verified to provide the best performance in terms of hysteresis response characteristics, they were selected in this parametric study for the strengthened beam and were denoted as BSA and BSB for the strengthened beams of groups A and B, respectively, and BUA and BUB for unstrengthened beams. The results of FEM of RC beams with different opening sizes are shown in Table 6.

**Figure 16:**
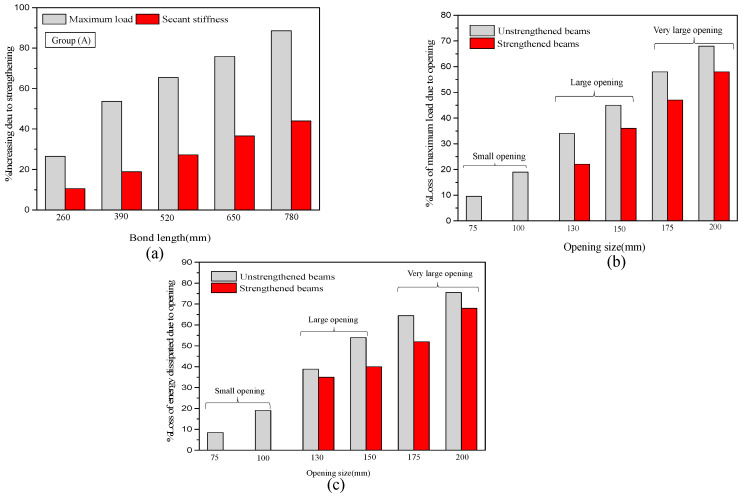
Effect of CFRP length and opening size on the performance of group A beams with respect to (**a**) secant stiffness, (**b**) maximum load and (**c**) energy dissipated at the ultimate state.

**Figure 17:**
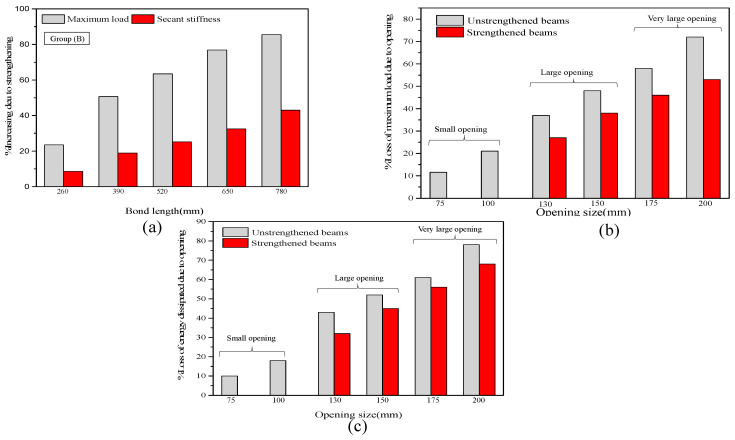
Effect of CFRP length and opening size on the performance of group B beams with respect to (**a**) secant stiffness, (**b**) maximum load and (**c**) energy dissipated at the ultimate state.

The analysis results of groups A and B clearly show that the proposed length of the CFRP sheet is successful in enhancing the maximum strength and secant stiffness with high efficiency. The shear crack propagated around the opening, in addition to there being improvements in energy absorption and the ductility ratio by gradually increasing the length. The two-layer U-warp with 780 mm length, which is equivalent to (6d), is the best among all lengths in enhancing the hysteretic characteristics of RC beams with openings. This finding could be because of the length of the FRP sheet, which added many confinement areas around the opening. The shear strength is enhanced at the opening location, thereby decreasing the appearance of brittle failure, particularly for group B beams. As shown in Table 6 and Figure 16a, the peak load increases by approximately 27% to 90%, and the effective stiffness increases by approximately 10% and 45% for group A beams (opening in the flexural zone) with a variable length of FRP sheet from minimum length to maximum (2d to 6d). In addition, a clear effect on the ductility of the specimen is observed, indicating an increase by approximately 11.65% to 82% compared with the unstrengthened specimen. As shown in Table 6 and Figure 17a, the percentage of increase in strength with respect to the maximum load and secant stiffness is approximately 23% to 86.5%, and the secant stiffness increases by 8% and 43.5% for group B beams. The effect on ductility is approximately 8.65% to 55%.

Figure 16 and Figure 17 show the effect of opening size on the performance of group A and B beams under cyclic loads. As shown in Table 6 and Figure 16 and Figure 17. The maximum load, effective stiffness, ductility and dissipated energy decrease with the increase in opening size. The flexural failure mode of the two groups was observed for unstrengthened beams with an opening diameter size of <40%. Depending on the classification of the circular web opening based on the ratio (circular diameter and overall depth) in terms of d/D as provided by Mansur [3], the percentage losses of maximum load and loss in dissipated energy caused by the opening were calculated for all unstrengthened and strengthened beams using the proposed maximum bond length of CFRP sheet beams and plotted versus the opening size ratio in a bar chart, as shown in Figure 16 and Figure 17. For group A and B beams with an opening of d/D < 40%, the reduction in maximum load ranges from 7% to 14.5%, and the loss in dissipated energy ranges from 6% to 16% for group A, while the percentage losses in maximum load and dissipated energy for group B beams (opening in the shear zone) are approximately 11% to 21% and 8.5% to 18%. For openings with d/D < 0.4, classified as ‘small’ openings, the percentage losses of maximum load and dissipated energy are less and within an acceptable range compared with the solid specimens (without openings). In this case, FRP strengthening may not be needed for the two groups to restore the original beam capacity. For the unstrengthened beams in the two groups with 0.4 < d/D < 0.6, the reduction in maximum load and dissipated energy ranges from 33% to 46% and 41% to 56% for group A and from 37% to 48% and 45% to 57% for group B, respectively, indicating that these beams are classified as ‘large’. In this case, the strengthened beam is effective in restoring the partial and original capacity. The decrease in loss caused by the strength effect ranges from 22.5% to 36% and 35% to 40% for group A and from 27% to 42%, and 36% to 47% for group B. For openings with d/D > 0.6, the loss in group A ranges from 58% to 70% and 66% to 76% and from 60% to 75% and 66% to 80% for group B, indicating that these beams are classified as ‘very large’. In this case, premature failure accrues because of the high shear stress concentration around the opening. Then, the FRP fails because of rupture. The result clearly shows that the strengthening efficiency immensely decreases and could only restore approximately 23% to 45% of the original stiffness for the two beam groups.

## 7. Conclusions

The major conclusions from the experiments of seven RC beams and 35 3D nonlinear FE models are presented as follows:

1. The results of the experiments and numerical simulations clearly show that beam capacity, stiffness degradation, energy absorption and failure mode of failure are significantly influenced by the openings located in the shear zone or flexural zone. The effectiveness of FRP strengthening in restoring the original capacity of the beam is confirmed. The similarity and good convergence between the experimental data and those from FE simulations prove that the identified model parameters are accurate, sensitive and acceptable in evaluating the strength and stiffness of beams with web openings. The effectiveness of FRP strengthening is verified, thereby demonstrating the feasibility of FEM in future research.
2. For the beam with an opening in the shear zone, the FRP sheet succeeds in improving the total strength by approximately 66.69%, which is considered the average result for the two orientations. In the flexural zone, the enhancement reaches 74.84% compared with the equivalent control beam, and the energy absorption increases by approximately 64.87% and 77.14%. Thus, the opening in the shear zone results in a remarkable decrease in the strength and stiffness failure of loads. A good correlation is obtained in the numerical and experimental results of hysteretic response, maximum load and damage mode, with an average correlation ratio of 90%.
3. The circular web openings for the two zones (bending and shear) of RC beams under cyclic loading can be categorized as small, large and very large on the basis of the d/D ratio in accordance with the classification conducted by Mansur [3], where d/D < 40% for small type and d/D > 50% for large type openings. The numerical simulation indicates that the recommended ratios of d/D < 40 as small, 0.4 < d/D < 0.6 as large and d/D > 0.6 as very large. This classification is in close agreement with the rectangular opening in the shear zone conducted by [23].
4. For small circular openings in the (shear/bending) zones of RC beams, the effect of opening size represents loss of strength, stiffness and energy absorption. The opening is remarkably smaller and within the appropriate range compared with that of solid beams. In this case, strengthening may be not needed. For large opening beams, FRP strengthening may be extremely important and used to restore the losses in beam capacity either fully or partially. The proposed increased bond length of two layers of FRP sheet (equivalent 2d) was used in this study and successfully restored the maximum load and energy absorption by approximately 77.5% and 65%, respectively, for the beam with an opening in the bending zone and by approximately 73% and 64% for the beam with an opening in the shear zone. For the very large opening, the external bonding of FRP is ineffective in restoring the original beam strength because of premature failure compression.

## Figures and Tables

**Table 1:** Test matrix.

Beam Name	Opening Size (mm)	FRP Schemes	FRP Organizing	Concrete Strength *f_c_* ^a^ (MPa)
B-0	-	-	-	30
BC-1	130	-	-	30
BC-2	130	-	-	30
BC-3	130	Vertical/Uwarp	2 layers (0/90)	30
BC-4	130	Inclined/Uwarp	2 layers (60/120)	30
BC-5	130	Vertical/Uwarp	2 layers (0/90)	30
BC-6	130	Inclined/Uwarp	2 layers (60/120)	30

^a^ Compressive strength of concrete.

**Table 2:** Material properties of steel reinforcement.

Grade	Grade	Diameter (Ø)	Yield Strength	Tensile Strength	Elongation
G.B ^1^	B.S ^2^	mm	MPa	MPa	%
HRB400	460	12	485.4	650	15
HRB400	460	10	480.3	570.5	15
HPB300	250	8	306.8	446.7	11

^1^ Chinese code of practices for mechanical properties of steel; ^2^ British standard requirements for steel properties.

**Table 3:** Properties of carbon fiber-reinforced polymer (CFRP).

Material	Tensile Strength (MPa)	Elastic Modulus (GPa)	Ultimate Strain (%)	Density (kg/m^3^)	Thickness (mm)
Carbon fiber	3400	230	≥1.6	1.72	0.335
CFSR-A *	40	2.40	≥1.5	-	-
CFSR-B *	38	2.40	≥1.5	-	-

Note: * refers to adhesive material A and material B; the mix proportion is 1:2, respectively.

**Table 4:** Key results of tested beams.

Specimen	Cracking Load (KN)	Pult ^1^ (KN)	Ymax ^2^ (mm)	Pmax ^3^ (KN)	Disp-Max ^4^ (mm)	Duc-Ratio ^5^ (μ)	Stiff-Ki ^6^ (kN/mm)	Failure Mode
B-0	42.55	95.12	11.38	118.92	27.43	2.38	10.44	Flexure
BC-1	38.75	79.07	10.53	98.84	25.78	2.16	9.28	Shear
BC-2	34.25	75.53	10.44	94.41	23.52	2.13	9.04	Flexure
BC-3	47.62	121.8	12.52	152.25	28.66	2.36	12.16	Flexure
BC-4	54.44	129.98	12.96	162.48	30.85	2.38	12.54	Flexure
BC-5	60.20	137.26	12.82	172.37	31.15	2.46	13.44	Flexure-shear
BC-6	61.75	138.59	12.76	173.24	32	2.55	13.58	Flexure-shear

^1^ Ultimate load; ^2^ yield displacement; ^3^ maximum strength; ^4^ maximum displacement; ^5^ ductility factor; ^6^ secant stiffness.

**Table 5:** Comparison of experimental and FE result for test beams.

EXP *	FE **	Pmax ^1^	Δmax ^2^	Duc-Ratio	Stiff-Ki	PEXP
Specimen	model	(KN)	(mm)	(μ)	(kN/mm)	PFE
B-0	M1	132.03	28.53	2.70	12.67	0.92
BC-1	M2	110.67	27.07	2.49	10.02	0.91
BC-2	M3	107.63	24.46	2.45	9.91	0.90
BC-3	M4	160.34	28.66	2.57	11.44	0.95
BC-4	M5	168.41	31.16	2.67	11.60	0.96
BC-5	M6	170.01	31.77	2.69	11.60	1.01
BC-6	M7	171.32	32.32	2.72	11.64	1.02

Note: EXP * refers to tested specimen; FE ** is equivalent for finite element model; ^1^ maximum load; ^2^ maximum displacement.

**Table 6:** Details and results of FE models used in the parametric study.

Beam Name	Bond Length of FRP (mm)	FRP Orientation	Opening Size (mm)	FE Results					
				Pmax ^h^	Δ-max ^i^	Y-max	Duc-Ratio	Stiff-Ki	Failure
				(KN)	(mm)	(mm)	(μ)	(KN/mm)	Mode
**Effect of FRP sheet parameters**									
B1-1 ^a^	260	Uwarp-2 layers *	130	140.23	26.05	12.02	2.21	10.67	Flexure
B1-2 ^a^	390	Uwarp-2 layers *	130	170.01	31.77	14.66	2.69	11.8	Flexure
B1-3 ^a^	520	Uwarp-2 layers *	130	182.23	33.55	15.72	2.87	12.58	Flexure
B1-4 ^a^	650	Uwarp-2 layers *	130	194.55	35.23	16.69	3.05	13.26	Flexure
B1-5 ^a^	780	Uwarp-2 layers *	130	208.05	36.34	17.8	3.23	14.14	Flexure
B2-1 ^b^	260	Uwarp-2 layers *	130	132.68	24.23	11.42	2.1	10.14	Shear
B2-2 ^b^	390	Uwarp-2 layers *	130	160.34	28.66	14.02	2.57	11.44	Y-sh **
B2-3 ^b^	520	Uwarp-2 layers *	130	174.24	31.87	14.77	2.76	12.12	Y-sh **
B2-4 ^b^	650	Uwarp-2 layers *	130	188.57	33.82	15.86	2.93	12.84	Flexure
B2-5 ^b^	780	Uwarp-2 layers *	130	200.15	34.28	16.91	3.07	13.57	Flexure
**Effect of opening diameter**									
B-0 ^c^		-	0	132.03	28.53	12.67	2.79	11.51	Flexure
BUA-75 ^d^	-	Un-strengthened	75	126.74	28.01	12.55	2.67	10.54	Flexure
BUA-100 ^d^	-	Un-strengthened	100	118.27	27.64	12.41	2.55	10.32	Flexure
BUA-130 ^d^	-	Un-strengthened	130	110.67	27.07	11.05	2.49	10.02	Flexure
BUB-75 ^e^	-	Un-strengthened	75	122.63	26.73	11.82	2.58	10.11	Flexure
BUB-100 ^e^	-	Un-strengthened	100	113.63	25.31	11.01	2.49	10.01	Flexure
BUB-130 ^e^	-	Un-strengthened	130	107.63	24.46	10.86	2.45	9.91	Flexure
BUA-150 ^e^	-	Un-strengthened	150	92.47	23.01	9.23	2.03	8.27	Y-sh **
BSA-150 ^f^	780	Uwarp-2 layers *	150	155.8	29.23	13.48	2.47	10.67	Y-sh **
BUA-170 ^d^	-	Un-strengthened	170	75.21	19.33	7.62	1.66	6.71	Y-sh **
BSA-170 ^f^	780	Uwarp-2 layers *	170	143.59	26.89	12.4	2.27	9.82	RP-at opening
BUA-200 ^d^	-	Un-strengthened	200	60.24	16.46	6.1	1.22	5.37	SF-at opening
BSA-200f ^f^	780	Uwarp-2 layers *	200	128.39	23.2	11.01	1.89	8.22	RP-at opening
BUB-150 ^e^	-	Un-strengthened	150	88.75	20.06	8.9	1.95	6.62	Y-sh **
BSB-150 ^g^	780	Uwarp-2 layers *	150	150.12	28.07	12.44	2.37	10.24	Y-sh **
BUB-170 ^e^	-	Un-strengthened	170	70.35	16.05	7.12	1.56	4.65	SF-at opening +
BSB-170 ^g^	780	Uwarp-2 layers *	170	136.91	26.68	11.42	2.13	9.21	RP-at opening =
BUB-200 ^e^	-	Un-strengthened	200	55.82	12.84	5.7	1.01	3.62	SF-at opening +
BSB-200 ^g^	780	Uwarp-2 layers *	200	122.61	20.23	9.92	1.34	6.27	RP-at opening +

Note: ^a,b^ refer to group A, B models to study the effects of bond length; ^c^ solid beam, model; ^d,e^ group A, B (unstrengthened) models to study the opening size; ^f,g^ group A, B (strengthened) models to study the opening size; ^h^ maximum load; ^i^ the maximum displacement; * vertical direction for CFRP schemes; ** yield at mid-region of the span and shear failure mode at near support; + shear failure at the opening; = the rupture in CFRP sheet.
